# Assessment of Ultra-Early Administration of Sacubitril Valsartan to Improve Cardiac Remodeling in Patients With Acute Myocardial Infarction Following Primary PCI: Rational and Design of a Prospective, Multicenter, Randomized Controlled Trial

**DOI:** 10.3389/fphys.2022.831212

**Published:** 2022-02-10

**Authors:** Zhengwei Li, Guosheng Fu

**Affiliations:** ^1^Department of Cardiology, Sir Run Run Shaw Hospital, Zhejiang University School of Medicine, Hangzhou, China; ^2^Key Laboratory of Cardiovascular Intervention and Regenerative Medicine of Zhejiang Province, Hangzhou, China

**Keywords:** acute myocardial infarction, sacubitril valsartan, cardiac remodeling, echocardiographic measurement, NT pro-BNP, cardiothoracic ratio (CTR)

## Abstract

**Background:**

Despite coronary re-vascularization, the common complications of acute myocardial infarction (AMI), cardiac remodeling, and heart failure (HF), is increasing globally. Sacubitril valsartan (SV), an angiotensin receptor-neprilysin inhibitor (ARNI), has been previously demonstrated to improve HF. We further hypothesize that ultra-early SV treatment is also effective in preventing cardiac remodeling for patients with AMI following primary percutaneous coronary intervention (PCI).

**Methods:**

The Assessment of ultra-early administration of Sacubitril Valsartan to improve cardiac remodeling in patients with Acute Myocardial Infarction following primary PCI (ASV-AMI) trial is a prospective, multicenter, randomized controlled trial in China planning to enroll at least 1,942 eligible patients from 10 centers. After successful primary PCI of culprit artery within 24 h, AMI patients are randomized to 2 h group or 3–7 days group with SV treatment. The major endpoints are echocardiographic measurement, cardiothoracic ratio, and N-Terminal pro-B-Type Natriuretic Peptide (NT pro-BNP) at baseline, 1, 3, 6, and 12 months. The secondary endpoints included MACE (cardiac arrest, cardiogenic death, myocardial infarction, and target vessel re-vascularization), in-/out-patient HF, EuroQol Five Dimensions Questionnaire (EQ-5D), and Kansas City Cardiomyopathy Questionnaire (KCCQ).

**Discussion:**

The ASV-AMI trial is the first clinical trial of ultra-early administration of SV in the treatment of post-PCI AMI, adding more clinical evidence. Early application of SV to prevent cardiac remodeling in AMI patient is a major focus of this trial.

**Clinical Trial Registration:**

Trial registration Chinese Clinical Trial Registry (http://www.chictr.org.cn; ChiCTR2100051979). Registered on 11 October 2021.

## Introduction

Acute myocardial infarction (AMI), including ST-segment elevated myocardial infarction (STEMI) and non-STEMI, remains a serious life-threatening event (Xu et al., 2020). The rate of death, heart failure (HF), and recurrent ischemic events occurring in the first years after myocardial infarction remains elevated in the high risk population (Ferreira et al., 2021). Despite coronary re-vascularization of culprit coronary artery, there is currently no excellent treatment for cardiac remodeling after coronary artery patency recovery. Growing evidence from clinical trials shows that AMI has a great impact on cardiac remodeling and HF (Duengen et al., 2020; Tripolt et al., 2020; Wang et al., 2020). According to Swede Heart Registration, heart failure after AMI accounted for 13–31% over 1 year (Desta et al., 2015). The risk of subsequent heart failure caused by cardiac remodeling is very high. It is urgent to optimize the management measures for high-risk AMI population in order to improve the prognosis.

Sacubitril valsartan (SV), an angiotensin receptor-neprilysin inhibitor (ARNI), has been previously demonstrated to improve cardiac remodeling and heart failure (Massimo et al., 2021a). The 2018 Chinese Heart Failure Diagnosis and Treatment Guidelines recommend that ARNI (Class I recommendation, Level B evidence) reduces the morbidity and mortality for heart failure with reduced ejection fraction (HFrEF). AMI, especially anterior wall MI, probably promotes the progress of cardiac remodeling. The Association of Change in N-Terminal pro-B-Type Natriuretic Peptide (NT pro-BNP) Following Initiation of Sacubitril-Valsartan Treatment With Cardiac Structure and Function in Patients With Heart Failure With Reduced Ejection Fraction (PROVE HF) trail reported that SV improved NT-proBNP concentrations, left ventricular ejection faction (LVEF), left ventricular end-diastolic volume index (LVEDVi), and left ventricular end-systolic volume index (LVESVi) in HFrEF patients (Januzzi et al., 2019). Available evidence may support SV as a first-line therapy in outpatient or in-hospital HFrEF patients (Massimo et al., 2021b). The Effect of Sacubitril-Valsartan vs. Enalapril on Aortic Stiffness in Patients With Heart Failure and Reduced Ejection Fraction: A Randomized Clinical Trial (EVALUATE-HF) trail found that SV improved left atrial volume index (LAVi), LVEDVi, and LVESVi compared with enalapril (Desai et al., 2019). So, ARNI has sufficient evidence to improve cardiac remodeling and clinic outcomes (Giovanna et al., 2020a).

Cardiac remolding is central to the progression of HF and occurs in response to AMI, which consists of the changes in cardiac geometry, function, or both, manifested by the reduced LVEF, LVEDVi, and LVESVi (Duengen et al., 2020). The pathological mechanism of the poor prognosis of AMI is cardiac remodeling, which often starts between 24 and 72 h after AMI. Cardiac remodeling is associated with risk of heart failure, cardiac death, and re-hospitalization, represented an important target for HF therapy. In the studies of guideline-directed medical therapies for HF, such as β-blocker, angiotensin-converting enzyme inhibitor (ACEI), angiotensin II receptor blocker (ARB), and mineralocorticoid receptor antagonist (MRA), increased LVEF, decreased LV volumes or both are associated with improved prognosis (Bao et al., 2021). In the process of cardiac remodeling post-AMI, we believe that the earlier we block the process of cardiac remodeling, the better the prognosis. We need a series of operation preparation post-PCI, such as random and drug preparation, so that our research can enter the next step. We choose 2 h post-PCI as our experimental group. However, the effects of SV on ultra-early preventing cardiac remodeling in patients with AMI remain unclear.

The pathophysiologic mechanism responsible for benefits of ARNI remains unclear. Neprilysin inhibition enhances circulating level of biologically active natriuretic peptides, which may have antihypertrophic, antifibrotic, and vasodilatory effects (Docherty et al., 2021). In PROVE HF and EVALUATE HF trails, the benefits of SV are associated with a decreased NT pro-BNP concentration, LVEDVi, LVESVi, cardiothoracic ratio, and increased LVEF. Reducted NT pro-BNP concentrations for HF is associated with reversed cardiac remodeling and HF. However, SV treatment has not established such a link. This trial examines the mechanism of the changes in NT pro-BNP concentrations, LVEF, LVEDVi, LVESVi, and cardiothoracic ratio after ultra-early administration of SV treatment in post-PCI AMI patients.

## Methods and Analysis

### Study Objective and Hypothesis

The objective of ASV-AMI is to test whether SV treatment prevents cardiac remodeling more effectively in 2 h group than in 3–7 days in post-PCI AMI patients. The working hypothesis is that SV improves LVEDVi, LVESVi, and NT-proBNP concentration in EVALUATE HF and PROVE HF trial. AMI stimulates the progression of cardiac remodeling and heart failure, which is demonstrated by the changes of echocardiographic parameters (LVEDVi, LVESVi, LAVi, E/e’, LVEDV, LVESV, LVEF, etc.), NT-proBNP concentrations, and cardiothoracic ratio.

The secondary objective is to study whether the effect of SV in 2 h group than in 3–7 days group is better in reducing in-/out-patient HF and major cardiovascular event (MACE), including cardiac arrest, cardiogenic death, myocardial infarction, and target vessel re-vascularization. The health quality of life is evaluated by EuroQol Five Dimensions Questionnaire (EQ-5D) and Kansas City Cardiomyopathy Questionnaire (KCCQ).

The primary safety assessment includes laboratory values, all adverse events within baseline, 1, 3, 6, and 12 months follow-up, hypotension, and vascular edema.

### Study Design

**Figure 1:**
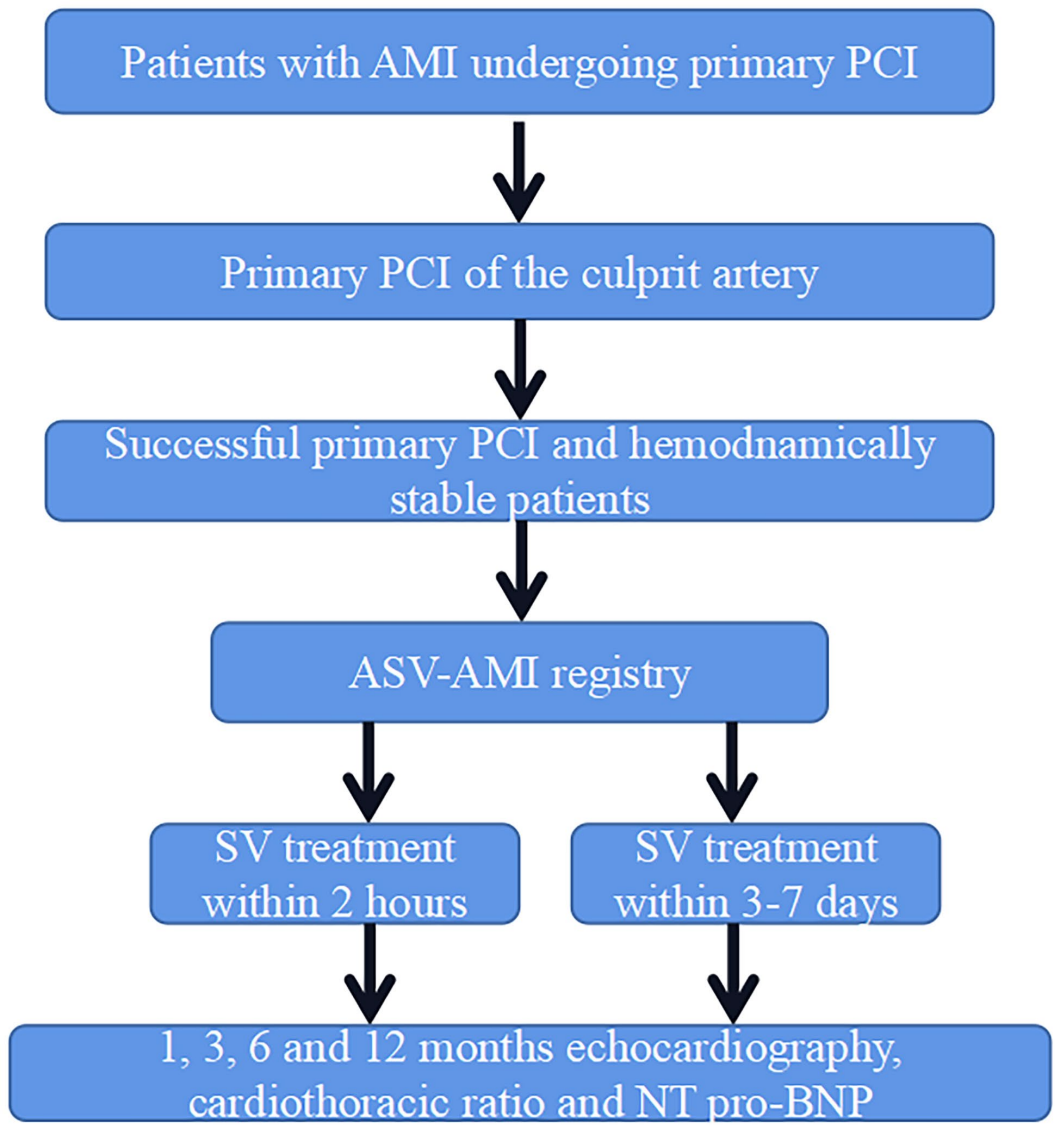
Study design.

This is a prospective, multicenter, randomized controlled trial in China. The steering and executive committee is responsible for scientific, operational, and medical process of the trial. The executive committee is also responsible for the integrity of data analysis and reporting results. The protocol has been approved by the Ethics Committee of Sir Run Run Shaw Hospital Affiliated to Zhejiang University School of Medicine (No. Keyan 20210907-9). ASV-AMI has been registered at www.chictr.org.cn (ChiCTR2100051979) on 11 October 2021. The trial starts recruitment in January 2022. The study flowchart is shown in Figure 1. Prior to the start of any trial procedure, each subject will be given written informed consent. In all, 1,942 patients are collected from 10 participating centers.

Sir Run Run Shaw Hospital Affiliated to Zhejiang University School of Medicine is the initiator of the investigator-initiated clinical trial. Department of cardiology in Sir Run Run Shaw Hospital is the clinical and data coordination center. Meanwhile, department of cardiology in Sir Run Run Shaw Hospital is responsible for the design and implementation, relevant statistical analysis, and preparation, including the drafting and editing of the document and its final version.

### Trial Population, Criteria, and Procedures

All patients aged 18 years or above with first-time or recurrent AMI undergoing primary PCI will be qualified by attending physicians. The PCI procedure will be carried out according to the standard procedures of the treatment hospital. The inclusion and exclusion of patients will be implemented by the attending physicians of PCI centers. After successful primary PCI of culprit artery, AMI patients are allocated to 2 h group or 3–7 days group after PCI. All other documented secondary therapeutic medication, including dual antiplatelet therapy, statins, β-blockers, etc., will be prescribed in accordance with existing guidelines judged by attending physicians.

The premise for participation is that there is no indication of cardiac shock, a past history of angioedema, bilateral renal artery stenosis, serum potassium > 5.2 mmol/L, estimating Glomerular Filtration Rate (eGFR) < 30 ml/min/1.73 m^2^, allergy to ARNI, ACEI, ARB, or other similar chemical drugs, any organ system in past 3 years and less than 1 year of life expectancy. Previous treatment with SV is not an exclusion criteria. Patients can participate in any other trial that does not directly alter the effect of SV or valsartan treatment. Further details on inclusion and exclusion criteria are summarized in Table 1.

**Table 1:** Key eligibility criteria for the ASV-acute myocardial infarction (AMI) study.

**Inclusion criteria**
Informed consent
Age ≥ 18 years
Patients with AMI presenting within 24 h of symptom onset
Successful primary PCI within 24 h of symptom onset
Medication within 24 h of symptom onset
**Exclusion criteria**
Cardiac arrest within 24 h
A history of angioedema
Known or suspected bilateral renal artery stenosis
eGFR < 30 ml/min/1.73 m^2^
Potassium > 5.2 mmol/L
A history of allergy to SV, ACEI, ARB, or NEP inhibitors or other similar chemical drugs
A history of malignancy in any organ system (except localized basal cell carcinoma of skin) in the past 3 years, with a life expectancy of less than 1 year
Patients are considered unsuitable to participate in the trial, including mental, behavioral, or cognitive impairment, which is enough to affect the ability of patients to understand, obey the protocol instructions, or complete the follow-up operation

### Interventions With Study Drug

If all eligibility criteria are met and written informed consent is provided, the patient will be registered as a SV treatment prescription. The dosage will be based on the clinical condition. Acceptable drugs and dosages include SV (400 mg daily total dose). Patients will be encouraged to continue taking SV until the end of the trial. Patients with hypotension after taking medication are advised to reduce or stop medication.

All study patients will receive an information letter to obtain and contact information about the sponsor from the local primary investigator and the central supervisor of Sir Run Run Shaw Hospital Affiliated to Zhejiang University School of Medicine. Patients will be contacted by a dedicated person to prevent medical contact or primary care visits that may affect treatment compliance.

### Data Collection and Monitoring

Detailed overview of data collected during follow-up is shown in Table 2. Baseline data will be obtained from hospital records and discharge letters (medication, comorbidity, cardiac rehabilitation, blood pressure, and weight and height measurements), echocardiography, and self-report EQ-5D and KCCQ. PCI hospitals will analyze the relevant standard blood samples (hematology, blood lipids, and clinical chemistry). Besides, blood samples will be sent to the central laboratory for NT-pro BNP concentrations test. The parameters of echocardiography will be analyzed in the core laboratory too. Standing chest radiographs are used to measure the cardiothoracic ratio.

**Table 2:** Data collection and schedule of assessments.

Phage	Screening				Treatment
Month	0	1	3	6	12
Informed consent	×				
Demographics	×				
Medical history	×				
Vital signs	×	×	×	×	×
Electrocardiogram	×	×	×	×	×
Myocardial enzymes	×	×	×	×	×
Stand chest X-ray	×	×	×	×	×
Myocardial enzymes	×	×	×	×	×
Echocardiography	×	×	×	×	×
NT pro-BNP	×	×	×	×	×
Killip/NYHA classification	×	×	×	×	×
Height	×				×
Weight	×				×
AE/SAEs		×	×	×	×
Biomarkers^*^	×			×	
Angioedema	×	×	×	×	×
EQ-5D^**^	×	×	×	×	×
KCCQ^***^	×	×	×	×	×
eCRF	×	×	×	×	×

* Hematology, clinical chemistry, and blood lipids.

** EuroQol Five Dimensions Questionnaire (EQ-5D).

*** Kansas City Cardiomyopathy Questionnaire (KCCQ).

An Case Report Form (CRF) will be provided for these patients to fill in at baseline, 1, 3, 6, and 12 months of follow-up. If there is a lack of responders, a reminder will be issued. EQ-5D and KCCQ will be provided and sent to patients. Vital signs and dosage of drugs are recorded during the follow-up. Each clinical trial site will be monitored by Office of Clinical Trial Institution, Sir Run Run Shaw Hospital Affiliated to Zhejiang University School of Medicine.

### Safe Monitoring and Reporting

After registration, all selected patients will be contacted by outpatient visits or telephone and interviewed on side effects after a standardized written agreement. The local PI will check the secure endpoint in the hospital records if patients do not response to the telephone call. Besides, PI of every center is responsible for reporting suspected serious adverse reactions and serious adverse events to the Data and Safety Monitoring Board according to Good Clinical Practice and the Office of Clinical Trial Institution, Sir Run Run Shaw Hospital Affiliated to Zhejiang University School of Medicine.

## Outcome Definitions and Measurements

The primary and secondary endpoints will be followed up for at least 1 year. Evaluation of main results will be obtained from echocardiography parameter, NT-pro BNP concentrations, and cardiothoracic ratio. Assessment of safety endpoints will be determined by administrative registries (adverse events, hyperkalemia, renal insufficiency, angioedema, and hypotension). All the registries have a reporting system and a unique personal identification number enabling us to link the participants to the registry.

## Sample Size Calculation and Statistic Assessment

Sample size is based on the available evidence published to date. We use https://www.cnstat.org/samplesize/4/ to generate the calculated sample size. We assume that baseline LVEDVi in 2 h group with SV was 70.3 ml/m^2^, while 75.6 ml/m^2^ in 3–7 days group used enalapril as a reference according to ELEVALUATE HF trial. The SD between the two groups was 37.6 ml/m^2^. Thus, patients in each group would at least offer 80% power at a one sided 0.05 significance level according to a 1:1 ratio of random group. Considering the maximum follow-up loss rate of 2.5% 1 year in both groups, it is anticipated that a total of 1,710 patients need to be recruited with 855 cases in each group. Based on the least squares mean of covariance model analysis, the treatment effect is estimated according to the ratio of the geometric mean with logarithmic baseline value as covariate and the bilateral 95% CI is reported. The analysis is based on all available data points assume that data is lost randomly.

The ASV-AMI trial is conducted by department of cardiology, Sir Run Run Shaw Hospital Affiliated to Zhejiang University School of Medicine. The ASV-AMI Steering Committee Overall is fully responsible for the overseeing independent academic researchers. The ASV-AMI Data and Safety Monitoring Board, which includes ACS experts and an independent statistician, actively monitors safety data, including all adverse and serious adverse events. The authors are fully responsible for the design and implementation of this trial, all research analysis, the drafting and editing of the trial, and its final content.

### Ethical Assessments

The ASV-AMI trial complies with the Declaration of Helsinki and Good Clinical Practice Guidelines. The scheme is independently approved by the institutional review committee of the participating center, and the written informed consent is obtained before registration. The trial protocol has been approved by the Ethics Committee of Sir Run Run Shaw Hospital Affiliated to Zhejiang University School of Medicine (No. Keyan 20210907-9) and registered at www.chictr.org.cn (ChiCTR2100051979) on 11 October 2020.

### Analysis

All data will be presented as percentages, mean ± SD or median (quartile range). Pearson correlation coefficients and the corresponding two-sided 95% credibility intervals (CIs) and *p* values will be calculated to examine the association between change in log-transformed NT-proBNP levels and each structural cardiac parameters (LVEDVi, LVESVi, LAVi, E/e’, LVEDV, LVESV, LVEF, and cardiothoracic ratio) from baseline to 1-, 3-, 6-, 12-month. The analyses will be repeated from baseline to 1-, 3-, 6-, 12-month. Sensitivity analyses will be performed using the last observation carried forward method used to impute missing data at 1 year.

An ANOVA will be conducted to compare the mean change in the EQ-5D and KCCQ scores between the two groups at baseline, 1-, 3-, 6-, 12-month. It is likely that groups defined in this way will be different with respect to characteristics that are also associated with the EQ-5D and KCCQ scores, including age (<75 vs. ≥75 years), LVEF (≤median vs. N median), and AMI types (STEMI vs. non-STEMI). Multivariable regression will be used to compare the mean change in EQ-5D and KCCQ scores by NT-proBNP and structural cardiac measurement groups, accounting for variability in individual patient characteristics by including them as covariates in the model. In addition, an optional sensitivity analysis using propensity score stratification (or matching) may be performed to provide an estimate of the differences by NT-pro BNP and structural cardiac measurement group between patients who are more similar with respect to baseline characteristics.

Pearson correlation co-efficients and their two-sided 95% CIs will be used to examine the association between both NT-proBNP and structural cardiac measurement and change from baseline. Echocardiographic variables will be calculated using values from follow up at baseline, 1-, 3-, 6-, 12-month.

### Ethics and Dissemination

The ASV-AMI trial is a prospective, multicenter, randomized controlled trial designed to assess the efficacy, safety, and tolerance of ultra-early SV treatment in post-PCI AMI. This is a vital clinical problem because of the lack of scientific evidence for the treatment of post-PCI AMI patients around the world. Although, it has been approved by China Food and Drug Administration and recommended by Chinese clinical guidelines in heart failure, the experience of SV administration in hospitalized AMI patients is limited. Meanwhile, it is well recognized that the results of initiating evidence-based drugs in hospital are consistent in greater long term. Considering the burden of cardiac remodeling and the unacceptably high incidence of post charge events, safely starting SV in this case may meet a vital unmet clinical need. The remarkable features of the ASV-AMI trial further strengthen the existing evidence base and collective understanding of ARNI in AMI and prevent cardiac remodeling in daily practice.

The ASV-AMI trial predominantly recruits AMI patients with primary PCI within 24 h of onset, requiring participants to take SV within 2 h or 3–7 days after PCI. Early cardiac remodeling often occurs in AMI within 24–72 h, including the infarct margin and distal non-infarcted myocardium. Cardiac remodeling includes cardiac hypertrophy and changes in ventricular structure to more evenly distribute increased wall stress, as extracellular matrix produces collagen scars to stabilize dilation and prevent more deformation (Rezq et al., 2021). Cardiac remodeling is determined by cardiac stretch, neurohormonal activation, paracrine and/or autocrine factors and activation of renin angiotensin aldosterone system (RAAS; Rouleau et al., 1993). The RAAS system is the main pathophysiological factor of cardiac remodeling. A large number of experiments have shown that RAAS inhibition can improve the prognosis of AMI (The Acute Infarction Ramipril Efficacy (AIRE) Study Investigators, 1993; Kober et al., 1995; Dickstein and Kjekshus, 2002; Velazquez et al., 2003). The natriuretic peptide system reverses the harmful effect of the RAAS upregulation on AMI, prevents the secretion of arginine and vasopressin and has a good regulatory on autonomic nervous system. Before early cardiac remodeling, AMI patients receive SV administration to prevent the progress of cardiac remodeling. This is the biggest highlight of this clinic trial.

In the early stage, oral administration was tried to inhibit neprilysin, which successfully elevated excretion of atrial natriuretic peptide (Gros et al., 1989). However, a study of long-term use of neprilysin inhibitor shows that the initial decline in blood pressure is not compulsorily. This may be due to the fact that neprilysin decomposes Angiotensin II (Hubers and Brown, 2016). Hence, in addition to increasing natriuretic peptides level, it can also increases angiotensin II level, which may counteract the effect of the former peptides. There is a strategy to eliminate the unnecessary effect of loneliness on neprilysin inhibition. ARNI can block the renin-angioten system and enhance the action of natriuretic peptide through angiotensin receptor antagonist, and ultimately prevent cardiac remodeling.

The Prospective comparison of ARNI with ACEI to Determine Impact on Global Mortality and morbidity in Heart Failure trial (PARADIGM-HF) is performed that SV is superior to enalapril in reducing the main endpoint of cardiovascular death and HF hospitalization (Mogensen et al., 2018). Compared with enalapril group, the risk of cardiac death and cardiac mortality in SV group is significantly lower, so the trial is terminated earlier. In fact, the reduction of HF mainly depends on cardiac remodeling. The clinical benefits of ACEI, ARB, β-blockers, and cardiac resynchronization therapy (CRT) are due to their effects on adaptive ventricular dilation and hypertrophy, as well as systolic dysfunction. According to the result of PARAMOUNT II trial, SV can significantly reduce the left atrial size and volume compared with valsartan (Solomon et al., 2012). Preclinical AMI trial also shows that neprilysin inhibitors can improve cardiac remodeling. In HF patients treated with SV, mitral regurgitation and left ventricular end diastolic filling volume are significantly improved. The results of PROVE-HF trail shows that the absolute increase of LVEF in patients with heart failure is 9.4%, which is related to the decrease of NT pro-BNP. These studies also suggest that the clinical benefit of SV may be related to its reversal cardiac remodeling. The latest PARADIGM-MI trial suggests that for patients who receive tablets from 12 h to 7 days (average 4.3 days) after the occurrence of AMI events, SV has a gradual improvement, and the longer the treatment time, the more benefits (Giovanna et al., 2020b). SV is better than ramipril in reducing the total event rate and the risk of main endpoint events reported by researchers (Khachfe et al., 2019). However, our experiment is more prospective. AMI patients start SV treatment much earlier in our trial. AMI patients undergoing primary PCI within 24 h of onset receives SV treatment within 2 h after PCI, which is the first known clinical trial of ultra-early SV administration in post-PCI AMI. This results in the prevention of cardiac remodeling at an ultra-early stage of AMI, which is the most brilliant innovation of this trial.

There are no data on the safety and efficacy of ARNI for AMI nowadays. According to our clinical conjecture, SV may improve the prognosis of AMI by preventing cardiac remodeling. RAAS/ARB therapy may be the standard treatment for AMI patients after PCI, which is one of the cornerstones of preventing cardiac remodeling after AMI. Compared with the standard ACEI/ARB regimens, the dual effect of inhibition of Angiotensin II receptors and neprilysin may play an important role in the prevention of cardiac remodeling after AMI.

As far as we know, this trial is the first clinical trial of ultra-early administration of SV in the prevention of cardiac remodeling post-PCI AMI. However, our study is not without limitations. This is a small-scale experiment with fewer events, so type 1 errors cannot be completely eliminated. In addition, our trial is not a multilateral trial, so the results may not be applicable to all post-PCI AMI patients in the worldwide. A larger ongoing randomized trial is waiting to confirm our results.

In conclusion, the ultra-early administration of SV in the treatment of post-PCI AMI may improve cardiac remodeling. Although this will add a new indication, this new drug needs to be further confirmed in a larger scale cohort of patients and follow up to ensure safety and efficacy for a longer time.

## Trial Status

The trial will be conducted according to the protocol version 1.0 of 11 October 2021. Patient recruitment started in January 2021 and is expected to run consecutively until December 2023. Data collection of the intervention phase is expected to be completed in January 2024 and data analysis in February 2024.

## Data Availability Statement

The raw data supporting the conclusions of this article will be made available by the authors, without undue reservation.

## Ethics Statement

The studies involving human participants were reviewed and approved by Ethics Committee of Sir Run Run Shaw Hospital Affiliated to Zhejiang University School of Medicine (Approved No. Keyan 20210907-9). The patients/participants provided their written informed consent to participate in this study.

## Author Contributions

ZL and GF were broadly involved in the conception and design of the study and drafted the manuscript. Furthermore, GF will be responsible for the logistic preparation and protocol-conform implementation of the study, the recruitment of patients, and the performance of training sessions. ZL critically reviewed the manuscript. The co-principal investigators of this study are ZL and GF. All authors have read and approved the final version of the manuscript and gave their consent for publishing this study protocol.

## Funding

This is an investigator-initiated trial supported by Sir Run Run Shaw Hospital Affiliated to Zhejiang University School of Medicine, which had no role in the design of the trial; preparation, review, or approval of the manuscript; or decision to submit the manuscript for publication. No funding was received for this trial.

## Conflict of Interest

The authors declare that the research was conducted in the absence of any commercial or financial relationships that could be construed as a potential conflict of interest.

## Publisher’s Note

All claims expressed in this article are solely those of the authors and do not necessarily represent those of their affiliated organizations, or those of the publisher, the editors and the reviewers. Any product that may be evaluated in this article, or claim that may be made by its manufacturer, is not guaranteed or endorsed by the publisher.
